# Experimental Validation of a Mathematical Framework to Simulate Antibiotics with Distinct Half-Lives Concurrently in an In Vitro Model

**DOI:** 10.3390/antibiotics10101256

**Published:** 2021-10-16

**Authors:** Brianna M. Eales, Cole S. Hudson, Iordanis Kesisoglou, Weiqun Wang, Michael Nikolaou, Vincent H. Tam

**Affiliations:** 1 Department of Pharmacological and Pharmaceutical Sciences, University of Houston, Houston, TX 77204, USA; bmeales@central.uh.edu (B.M.E.); cshudson217@gmail.com (C.S.H.); wwang6@central.uh.edu (W.W.); 2 Department of Chemical and Biomolecular Engineering, University of Houston, Houston, TX 77204, USA; akiskess@gmail.com (I.K.); Nikolaou@uh.edu (M.N.); 3 Department of Pharmacy Practice and Translational Research, University of Houston, Houston, TX 77204, USA

**Keywords:** pharmacokinetics, pharmacodynamics, experimental therapeutics, antimicrobial agents

## Abstract

Antimicrobial resistance has been steadily increasing in prevalence, and combination therapy is commonly used to treat infections due to multidrug resistant bacteria. Under certain circumstances, combination therapy of three or more drugs may be necessary, which makes it necessary to simulate the pharmacokinetic profiles of more than two drugs concurrently in vitro. Recently, a general theoretical framework was developed to simulate three drugs with distinctly different half-lives. The objective of the study was to experimentally validate the theoretical model. Clinically relevant exposures of meropenem, ceftazidime, and ceftriaxone were simulated concurrently in a hollow-fiber infection model, with the corresponding half-lives of 1, 2.5, and 8 h, respectively. Serial samples were obtained over 24 h and drug concentrations were assayed using validated LC-MS/MS methods. A one-compartment model with zero-order input was used to characterize the observed concentration-time profiles. The experimentally observed half-lives corresponding to exponential decline of all three drugs were in good agreement with the respective values anticipated at the experiment design stage. These results were reproducible when the experiment was repeated on a different day. The validated benchtop setup can be used as a more flexible preclinical tool to explore the effectiveness of various drug combinations against multidrug resistant bacteria.

## 1. Introduction

The widespread use of antibiotics for decades has led to the emergence of antimicrobial resistance, which presents a significant threat to global public health. In 2019, the CDC reported that there are over 2.8 million infections and 35,000 deaths from antibiotic-resistant bacteria each year in the U.S. alone [1]. It is predicted that, in 30 years, antibiotic-resistant infections will be the leading cause of death globally [2]. Infections caused by many multidrug-resistant bacteria are now untreatable because effective antibiotics are not available. The development of new antibiotics has not kept pace with the rate which bacteria are developing resistance to antibiotics currently available. Experts at the CDC are calling this the “post-antibiotic era” and are urging the development of new therapies capable of treating antibiotic-resistant infections [1].

The in vitro activity against a pathogen over time is most commonly determined using constant concentrations of an antibiotic (i.e., static models). By using constant dilution of an isovolumetric system and repeated dosing, a fluctuating concentration-time profile mimicking clinical dosing in humans can be produced (i.e., dynamic models). This would allow more clinically relevant insights to be gained, guiding early drug development and transition to in vivo/clinical investigations [3]. With a more sophisticated experimental setup, the dynamic model can be further modified to mimic two antibiotic exposures simultaneously, where the elimination half-lives of the individual antibiotics are distinctively different [4]. This framework has been the “gold standard” of the field for more than three decades, and has been used by multiple investigator groups to evaluate combination therapy [5,6,7,8,9,10,11,12,13,14,15,16,17].

With an increasing demand of testing antibiotic combinations against multidrug resistant pathogens, a more advanced experimental setup is necessary. Multiple (three or more) antibiotics with different mechanisms of action and elimination half-lives may have to be used together in unique situations. We have previously reported a mathematical framework to mimic the concentration-time profiles of more than two antibiotics simultaneously [18], which significantly extended the prior “gold standard” for two antibiotics. For three antibiotics with different elimination half-lives, two experimental designs (i.e., serial or parallel) are possible. The serial design was experimentally validated earlier. In this study, we report the experimental validation of the parallel design. For the purpose of illustration, the pharmacokinetics of meropenem, ceftazidime, and ceftriaxone were simulated concurrently in an in vitro hollow-fiber infection model.

## 2. Results

### 2.1. Ceftriaxone Assay Performance

**Figure 1:**
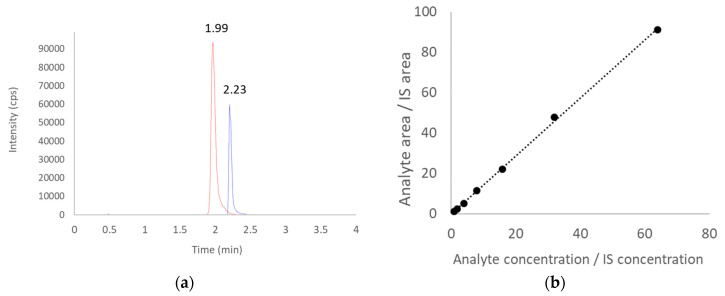
Performance of the ceftriaxone assay showing: (**a**) Elution of ceftriaxone (red) and ertapenem (blue, IS—internal standard) in a representative chromatogram; (**b**) Linear range of the mass spectrometry signals (r^2^ = 0.999).

The performance of the modified assay was deemed satisfactory. A typical chromatogram and the linear range of the assay are both shown in Figure 1. By using standard concentrations of 1, 8, 64 mg/L, the intra-day and inter-day variability were <3.9% and <5.6%, respectively.

### 2.2. Pharmacokinetic Simulations

**Figure 2:**
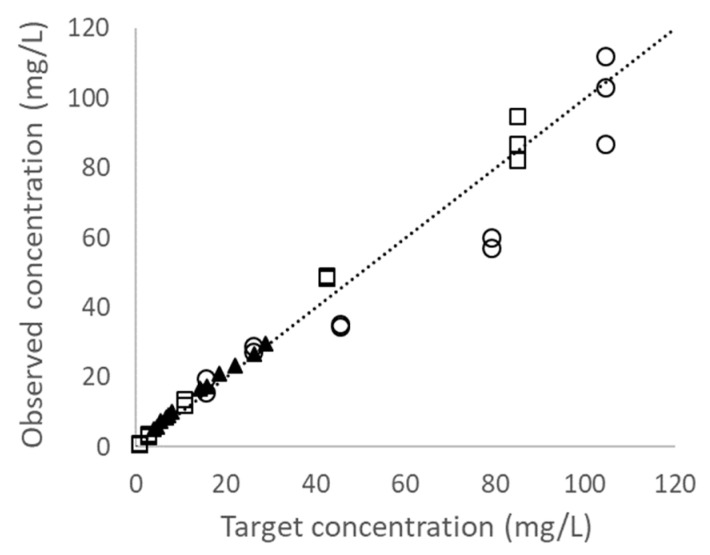
Correlation between target vs. observed antibiotic concentrations (r^2^ = 0.959): meropenem (open squares); ceftazidime (open circles); ceftriaxone (solid triangles). The dashed line depicts the line of identity, y = x. The equation for the line of best-fit is: Observed = 0.924 × Target + 2.118.

**Figure 3:**
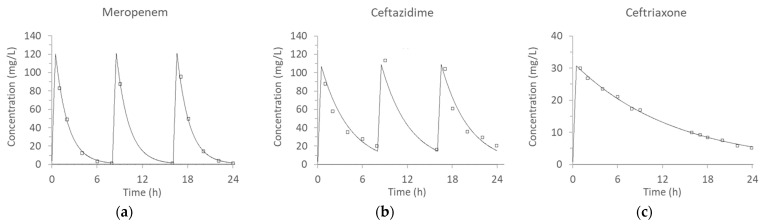
Concurrent concentration-time profiles of different antibiotics: (**a**) meropenem (half-life = 1.1 h, r^2^ = 0.995); (**b**) ceftazidime (half-life = 2.6 h, r^2^ = 0.926); (**c**) ceftriaxone (half-life = 9.4 h, r^2^ = 0.998). Open symbols represent experimental observations, and solid lines depict the best-fit models.

Individual drug concentrations observed ranged from 72% to 135% (median 112%) of the target values. The correlation between target vs. observed antibiotic concentrations is shown in Figure 2. The concentration-time profiles observed are shown in Figure 3. The estimated area under the curve over 24 h (AUC_24_) for meropenem, ceftazidime, and ceftriaxone were 674.8, 1125.0, and 347.2 mg·h/L, respectively (all within 30% of targets). Overall, the concurrent pharmacokinetic simulations were considered acceptable. The results were also reasonably reproducible on different days. The 80% confidence intervals of half-life observed on different days ranged from 0.83–1.3 h (meropenem), 0.99–3.6 h (ceftazidime), and 7.5–10.6 h (ceftriaxone), respectively (data not shown).

## 3. Discussion

Novel treatment strategies are needed to solve an urgent healthcare crisis with broad spectrum antimicrobial resistance. Antimicrobial agent combinations are clinically used in the routine care of patients with HIV and tuberculosis infections. Relatively speaking, antibiotic combinations are not considered as the standard of care for many bacterial infections. However, with a growing prevalence of infections due to multidrug-resistant bacteria, antibiotic combinations are increasingly used by clinicians as a treatment of last resort. A robust system to evaluate the efficacy of antibiotic combinations in vitro would be of great value to advance medical care of these patients. Until recently, simulating more than two distinct pharmacokinetic profiles simultaneously in a dynamic infection model would not have been feasible. This is generally recognized as a technical gap of the field.

To simulate antibiotic exposures with three distinct elimination half-lives, we reported two feasible experimental designs (i.e., serial or parallel). Both designs are based on the concept of using supplemental dosing (i.e., dosing in the supplemental tank(s)) to offset the rapid clearance implemented for the antibiotic with the shortest half-life. Theoretical details of the concept and complete mathematical formulas for design of the experimental setup have been described elsewhere [18]. Doses to be given are to achieve the expected maximum concentration (C_max_) by simple proportions (i.e., C_max_ = dose/volume), and the dosing frequencies are to emulate those of the clinical dosing regimens. The serial design is easier to set up, and it has been experimentally validated earlier [18]. On the other hand, the parallel design is more flexible and would allow three or more antibiotics to be simulated concurrently with minimal modifications in experimental setup. The parallel design can thus be considered as a step forward (from our previous serial design work) towards an even higher level of simulation complexity and flexibility. In this study, we focused on the experimental valuation of three antibiotics using the parallel design. A constant dilution rate to different supplemental volumes (i.e., CL_MEM_ = CL_CAZ_ = CL_CRO_) was used in this study. Notably, different dilution rates to a constant supplemental volume (for each antibiotic) are also possible, depending on investigator preference and equipment availability.

The simulated pharmacokinetic profiles were reasonably satisfactory. Despite a considerable difference (i.e., 8-fold), the best-fit half-lives of all three drugs were within the target ranges. Furthermore, these observations were fairly consistent when the experiment was performed on different days. The efficacy of specific antibiotic combination(s) against multidrug resistant bacteria is beyond the scope of this study, but it will be the focus of future studies once this pharmacokinetic simulation framework is established.

## 4. Materials and Methods

### 4.1. Antibiotics and Chemical Reagents

Meropenem and ceftazidime powder were obtained from Tokyo Chemical Industry Co., Ltd. (TCI) (Portland, OR, USA). Ceftriaxone powder was purchased from Sigma-Aldrich, Inc. (St. Louis, MO, USA). Ertapenem was provided by Merck (Rahway, NJ, USA). Ammonium acetate was purchased from Sigma Life Sciences (St. Louis, MO, USA). LCMS-grade ammonium hydroxide, water, and methanol were obtained from EMD Millipore Corporation (Billerica, MA, USA).

### 4.2. Experimental Setup

**Figure 4:**
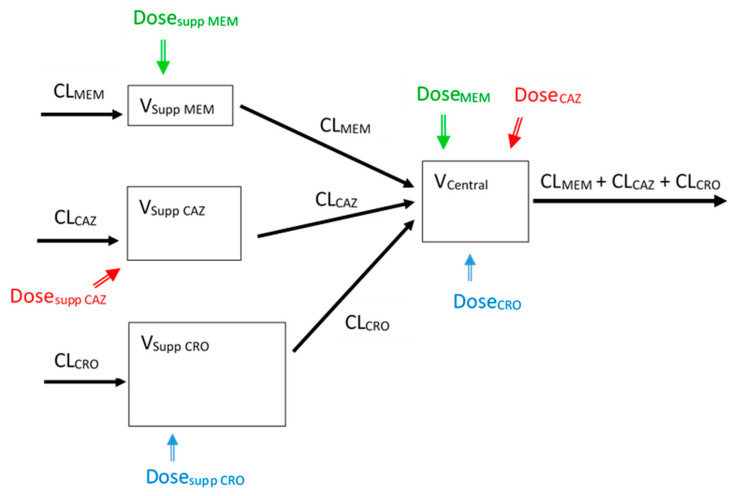
Schematics of the experimental setup. MEM—meropenem, CAZ—ceftazidime, CRO—ceftriaxone, CL—clearance, V—volume, supp—supplemental; CL_MEM_ = CL_CAZ_ = CL_CRO_ = 0.7 mL/min; V_central_ = 180 mL (includes connecting tubings and the hollow fiber cartridge), V_supp MEM_ = 60.6 mL, V_supp CAZ_ = 151.5 mL, V_supp CRO_ = 484.7 mL; Dose_MEM_ = 21,600 µg, Dose_CAZ_ = 21,600 µg, Dose_CRO_ = 5400 µg; Dose_suppMEM_ = 214 µg, Dose_suppCAZ_ = 32,934 µg, Dose_suppCRO_ = 38,227 µg.

The schematics of the experimental setup (parallel design) is shown in Figure 4. Target maximum concentrations in the central compartment (V_central_) were achieved by direct drug injections (Dose_MEM_, Dose_CAZ_, and Dose_CRO_). Diluent was introduced to the central compartment to dilute the drugs over time at pre-determined rates; an equal volume of drug-containing fluid was removed from the central compartment to maintain an iso-volumetric system. To mimic different pharmacokinetic exposures concurrently, dosing (Dose_supp_) into various supplemental compartments (V_supp_) would also be necessary. Meropenem and ceftazidime were dosed once every eight hours, whereas ceftriaxone was dosed only once to be consistent with clinical dosing in humans. All doses were given over 30 min. 

Specific pharmacokinetic parameters to mimic the unbound concentration-time profiles associated with clinical dosing in humans are shown in Table 1. To validate the pharmacokinetic profiles, serially samples (500 µL) were obtained from the circulatory loop (part of the central volume) in duplicate 1, 2, 4, 6, 8 (pre-dose), 9, 16 (pre-dose), 17, 18, 20, 22, and 24 h after the first doses were given. The samples were stored at −80 °C until drug analysis. The experiment was repeated once on a different day.

### 4.3. Drug Assays and Pharmacokinetic Modeling

Antibiotic concentrations in the samples were assayed using validated liquid chromatography tandem mass spectroscopy (LC-MS/MS) methods. The method used to detect meropenem and ceftazidime has been reported previously [19]. Using standard concentrations of meropenem (2, 8, 32, and 128 mg/L), the intra-day and inter-day variability were <6.9% and <11.6%, respectively. In addition, using standard concentrations of ceftazidime (2, 8, 32, and 128 mg/L), the intra-day and inter-day variability were <7.3% and <8.7%, respectively.

A modified method was used to assay ceftriaxone. Briefly, 20 μL of thawed sample was added to 880 μL of water and 100 μL of ertapenem at a concentration of 320 ng/mL as the internal standard. The samples were mixed by vortexing for 10 s before injection. The LC-MS/MS system consisted of an Exion UHPLC (SCIEX, Framingham, MA, USA) with a Kinetex 100 × 2.1 mm, a 5-μm EVO C18 100 Å column (Phenomenex, Torrance, CA, USA), and a QTRAP^®^ 5500 mass spectrometer (SCIEX, Framingham, MA, USA) equipped with Turbo-Ion-Spray™ source. Mobile phase A consisted of 10 mM ammonium acetate and 0.2% (*v*/*v*) ammonium hydroxide. Mobile phase B was 100% methanol. The injection volume for ceftriaxone was 5 µL. The analyte was separated by a gradient elution at 45 °C, using a flow rate of 0.3 mL/min. The gradient was as follows: 0–0.5 min, 95% A; 0.5–2.5 min, 95–10% A; 2.5–4.0 min, 10% A; and 4.0–4.2, min 10–95% A. Multiple reaction monitoring (MRM) scan type in positive mode was used for the mass spectrum. The transitions of m/z 555.1→396.1 for ceftriaxone and 476.2→432.2 for ertapenem were used. The best-fit weighing was used to calculate the slope, intercept, and correlation coefficient of the linear regression equation. The linear range of the assay is 1–64 mg/L.

To characterize the pharmacokinetics, a one-compartmental model with zero-order infusion input was fitted to the observed drug concentration-time profiles using ADAPT 5 [20]. The area under the curve over 24 h (AUC_24_) was derived by integrating the best-fit concentration-time profile with respect to time. The simulated concentration-time profiles were considered acceptable if the best-fit maximum concentrations and half-lives were both within 20% of target values.

## 5. Conclusions

Concurrent simulations of 3 clinically relevant antibiotic exposures were experimentally validated in a pre-clinical infection model. This framework could advance the field of antimicrobial pharmacokinetics/pharmacodynamics, and facilitate the in vitro efficacy of additional antibiotic combinations to be investigated against different pathogens. Future studies will extend the framework to four or more agents with different elimination half-lives.

## Figures and Tables

**Table 1:** Target pharmacokinetics and equivalent dosing in humans.

Antibiotics	C_max_ (mg/L) ^1^	Half-Life (h)	AUC_24_ (mg·h/L) ^2^	Equivalent Human Dosing
Meropenem	120	1.0	519.5	2 g
Ceftazidime	120	2.5	1298.7	2 g
Ceftriaxone	30	8.0	346.3	2 g

^1^ Unbound maximum concentration. ^2^ Meropenem and ceftazidime dosed every eight hours, ceftriaxone dosed once daily.
